# Rift Valley Fever Virus Is Lethal in Different Inbred Mouse Strains Independent of Sex

**DOI:** 10.3389/fmicb.2020.01962

**Published:** 2020-08-21

**Authors:** Haley N. Cartwright, Dominique J. Barbeau, Anita K. McElroy

**Affiliations:** ^1^ Division of Pediatric Infectious Disease, Department of Pediatrics, University of Pittsburgh, Pittsburgh, PA, United States; ^2^ Center for Vaccine Research, University of Pittsburgh, Pittsburgh, PA, United States

**Keywords:** Rift Valley fever virus, mouse models, inbred mice, sex, recombinant, viral hepatitis

## Abstract

Rift Valley fever virus (RVFV) is a zoonotic arbovirus affecting humans and livestock in Africa and the Arabian Peninsula. The majority of human cases are mild and self-limiting; however, severe cases can result in hepatitis, encephalitis, or hemorrhagic fever. There is a lack of immunocompetent mouse models that faithfully recapitulate the varied clinical outcomes of RVF in humans. However, there are easily accessible and commonly used inbred mouse strains that have never been challenged with wild-type RVFV. Here, RVFV susceptibility and pathogenesis were evaluated across five commonly used inbred laboratory mouse strains: C57BL/6J, 129S1/SvlmJ, NOD/ShiLtJ, A/J, and NZO/HILtJ. Comparisons between different mouse strains, challenge doses, and sexes revealed exquisite susceptibility to wild-type RVFV in an almost uniform manner. Never before challenged NOD/ShiLtJ, A/J, and NZO/HILtJ mice showed similar phenotypes of Rift Valley fever disease as previously tested inbred mouse strains. The majority of infected mice died or were euthanized by day 5 post-infection due to overwhelming hepatic disease as evidenced by gross liver pathology and high viral RNA loads in the liver. Mice surviving past day 6 across all strains succumbed to late-onset encephalitis. Remarkably, sex was not found to impact survival or viral load and showed only modest effect on time to death and weight loss for any of the challenged mouse strains following RVFV infection. Regardless of sex, these inbred mouse strains displayed extreme susceptibility to wild-type RVFV down to one virus particle.

## Introduction

Rift Valley fever (RVF) is a disease of humans and livestock causing severe economic and human health impacts (Hartman, 2017). Outbreaks of veterinary and human RVF occur throughout the Middle East and Africa with serosurveys indicating widespread human infection (LaBeaud et al., 2007, 2008, 2011). In people, RVF spans a variety of clinical manifestations, ranging from an acute flu-like illness to a more severe and sometimes lethal form of disease (Laughlin et al., 1979). The vast majority of human RVF virus (RVFV) infections result in self-limited febrile illnesses, but 10–20% of identified human cases progress to severe hepatitis, hemorrhagic fever, or encephalitis (Laughlin et al., 1978; Strausbaugh et al., 1978; Nanyingi et al., 2015). These large variations in human RVF disease progression and outcome are inadequately represented in the current small animal models. This lack of accurate recapitulation of human disease continues to limit our understanding of RVFV pathogenesis.

Currently, the most faithful recapitulation of human disease is displayed in various non-human primate models (Peters and Linthicum, 1994; Ross et al., 2012; Lorenzo et al., 2015). However, the use of non-human primate models is not feasible for large-scale or high-throughput studies. To date, all tested inbred mouse models are highly susceptible to wild-type RVFV and nearly all die of severe and early-onset hepatic disease. A major exception to this are BALB/c mice that live longer following infection and are more prone to develop neurological disease (do Valle et al., 2010; Smith et al., 2010; Reed et al., 2013; Lathan et al., 2017). On the other side of the spectrum exist the highly susceptible MBT and C57BL/6J mice that exhibit 100% mortality in 3–4 days (do Valle et al., 2010; Gray et al., 2012; Lathan et al., 2017). Although these models demonstrate the existence of some variation in RVFV disease outcome in inbred mice, they are still overwhelmingly lethal unlike human RVFV disease. Additionally, they only recapitulate severe hepatitis and inconsistently display late-onset encephalitis. However, the notable differences in survival times and disease skewing between these strains do point to the possibility of identifying mice with additional RVFV phenotypes.

Inbred rats have shown impressive differences in disease susceptibility to RVFV between strains. RVFV infection in rats ranges from extreme lethality in the highly susceptible Wistar-Furth (WF) rat to a complete absence of symptoms or death in subcutaneously infected Lewis rats (Peters and Anderson, 1981; Peters and Slone, 1982; Anderson et al., 1987). Distinct clinical outcomes of RVFV infection also exist between inbred rat models, with WF rats dying of acute hepatitis while August-Copenhagen-Irish (ACI) rats die of a late-onset encephalitic disease (Bucci et al., 1981; Peters and Anderson, 1981; Peters and Slone, 1982; Anderson et al., 1987). Interestingly, upon aerosol challenge, Lewis rats display a distinct phenotype, succumbing almost uniformly to encephalitis (Bales et al., 2012).

Due to these divergent clinical outcomes from RVFV infection in both mice and rats, we deemed it useful to investigate other classically used inbred mouse strains for their susceptibility to and disease manifestations of RVFV. Three out of five of the chosen strains for this study had never been investigated in the context of wild-type RVFV infection and the 129S1/SvlmJ strain had only briefly been studied (do Valle et al., 2010). Using the C57BL/6J mouse genome as a reference, the other four selected inbred mouse strains (129S1/SvlmJ, NOD/ShiLtJ, A/J, and NZO/HILtJ) vary at 4 million single nucleotide polymorphisms (SNPs) (Keane et al., 2011). Due to this existing genetic variability between strains, these inbred mouse strains have been able to capture a range of human disease manifestations for other viral infections. Leist et al. (2016) discovered highly variable disease phenotypes, including survival, body weight, and viral load, across investigated mouse strains with even inbred strains showing clear divergence in their susceptibility to H3N2 infection. Significant differences in SARS-CoV pathogenesis and disease severity were also found by Gralinski et al. (2015) upon challenge of various inbred and outbred mice. While this paper found fascinating differences in the outbred mouse resource, they were even able to identify an expansion of SARS-CoV phenotypes within common inbred mouse strains.

The study described here was undertaken to assess the susceptibility of five commonly used inbred laboratory mouse strains to wild-type RVFV. We investigated mouse strain, viral dose, sex, weight loss, and viral load following challenge with the wild-type ZH501 strain of RVFV. This report presents evidence for the overwhelming lethality of wild-type RVFV, down to a single virion, across C57BL/6J, 129S1/SvlmJ, NOD/ShiLtJ, A/J, and NZO/HILtJ inbred mouse strains, independent of sex.

## Materials and Methods

### Ethics Statement and Biosafety Information

All research in this study was conducted under the oversight of the University of Pittsburgh IACUC (protocol 19044158). All experiments with wild-type RVFV ZH501 strain were performed in the University of Pittsburgh Regional Biocontainment Laboratory (RBL) Biosafety Level 3 (BSL-3) and Animal Biosafety Level 3 (ABSL-3) facilities.

### Virus Generation, Growth, and Titer

The wild-type ZH501 strain of RVFV was originally isolated from a febrile human during the 1977 Egyptian epidemic (Meegan, 1979). For this study, recombinant wild-type RVFV was generated using an established reverse-genetics system based on the ZH501 strain background (Bird et al., 2007a; Gerrard et al., 2007). Virus stocks were fully sequence confirmed using next generation sequencing prior to use. Viral titer of the passage two stock was determined using a standard tissue culture infective dose 50 (TCID_50_) assay. Briefly, viral stocks were serially diluted in Dulbecco’s Modified Eagle Medium (DMEM) then added to 96-well plates containing 1 × 10^4^ Vero-E6 cells/well (eight replicates per viral dilution). Titers were determined through visualization by indirect fluorescent antibody assay (IFA) using a 1:500 dilution of a custom RVFV anti-N polyclonal #5584 (Genscript) as primary antibody and a 1:500 dilution of anti-rabbit 488 (Invitrogen) as secondary antibody. TCID_50_ titers were calculated using the Reed and Muench calculations (Reed and Muench, 1938).

### Mouse Study Design

Six to eight-week-old female and male A/J (stock #000646), C57BL/6J (stock #000664), 129S1/SvlmJ (stock #002448), NOD/ShiLtJ (stock #001976), and NZO/HILtJ (stock #002105) inbred mice were purchased from Jackson Laboratories. All mice were housed in HEPA filtration racks in the RBL’s ABSL-3 facility and provided *ad lib* access to food and water. All mice were infected with recombinant wild-type RVFV ZH501 strain under isoflurane anesthesia *via* left rear footpad (FP) injection to imitate a mosquito bite. Viral infection doses in these studies ranged from 0.2 to 2,000 TCID_50_ per animal, which equates to doses ranging from 0.138 to 1,380 PFU per animal (0.69 TCID50 = 1 PFU; Poisson distribution based upon Reed and Muench, 1938). Mice received a 20 μl injection of virus diluted in sterile phosphate buffered saline (PBS). For all experiments, daily weights were recorded, and mice were evaluated at least once daily for clinical signs of disease. Mice were euthanized according to a predetermined clinical scoring method (Supplementary Table S1). At the time of euthanasia, mice were anesthetized with isoflurane and blood was collected *via* cardiac puncture. Following cervical dislocation, liver, spleen, brain, and testes (where applicable) were collected for subsequent RNA extraction and viral RNA load quantitation.

### RNA Extraction and Quantitative RT-PCR

Mouse liver, spleen, brain, and testes tissue samples were weighed and then homogenized in sterile PBS with 1X Antibiotic-Antimycotic (Gibco) using a D2400 Homogenizer (Benchmark Scientific). RNA was extracted from liver, spleen, brain, and testes tissue samples with TRIzol reagent (Ambion) following the Direct-zol RNA purification protocol (Zymo Research). Quantitative RT-PCR (qRT-PCR) targeting the L segment of RVFV (Bird et al., 2007b) was performed using the SuperScript III Platinum One-Step qRT-PCR kit (Thermofisher). T7 driven RVFV L RNA template of known quantity was serially diluted to generate a RVFV RNA standard curve. This template RNA was made by In-Fusion cloning (Takara Bio), a fragment of the ZH501 RVFV L segment into pET-9a (Millipore Sigma). To generate linear pET-9a primers 5'-AATCCTCAAACTTCTGGGAAACCGTTGTGGTC-3' and 5'-TTCAAAGCTTATCATTCTAGAAATAATTTTGTTTAACTTTAAGAAGGA-3' were used. To prepare the RVFV L segment fragment primers 5'-AAAATTATTTCTAGAATGATAAGCTTTGAAGAGATCCAT-3' and 5'-CCACAACGGTTTCCCAGAAGTTTGAGGATTGTATGAGG-3' were used. The resultant plasmid, pLquant, was gel purified and linearized with XbaI (New England Biolabs), and then used as template in a TranscriptAid T7 High Yield *in vitro* transcription reaction (Thermo Scientific). Product RNA was purified using the GeneJET RNA Purification kit (Thermo Scientific), and then diluted to known copies/ml in RNase-free water for use as qRT-PCR standard curve template. The assay was performed using a C1000 Touch Thermo Cycler/CFX96 Real-Time System (Bio-Rad) under the following reaction conditions: 50°C for 15 min, 95°C for 3 min, and then 40 cycles of 95°C for 15 s and 55°C for 1 min. RNA copies for each unknown sample were normalized by tissue weight and are reported as log viral RNA copies per milligram of tissue. The lowest limit of detection of this assay was calculated as the highest Ct value detected in the standard curve multiplied by 50 to account for dilutions, and then divided by the average of all sampled tissue weights in mg.

### Enzyme-Linked Immunosorbent Assay

MaxiSorp plates (Thermo Scientific) were coated with lysate, diluted 1:1,000 in PBS, from RVFV-infected Vero-E6 cells or with lysate from uninfected Vero-E6 cells to act as a negative control (McElroy et al., 2009). Plates were left at 4°C overnight, and then blocked in blocking buffer (5% non-fat milk in PBS-0.1% Tween 20) at 37°C for 1 h. Terminal mouse serum samples were serially diluted in blocking buffer, and then incubated on blocked plates at 37°C for 2 h. All serum samples were assayed in duplicate alongside normal mouse serum as a negative control. After incubation with sera, plates were washed three times with PBS-0.1% Tween 20 (PBST), and then incubated for 1 h at 37°C in anti-mouse IgG-HRP (Jackson ImmunoResearch) diluted 1:5,000 in blocking buffer. Following three PBST washes, the plates were incubated in tetramethylbenzidine (TMB) substrate, and then stopped with TMB stop solution. Plates were read at 450 nm and the raw data were analyzed in Excel by subtracting the negative control absolute values from those of the RVFV lysate plate. The endpoint ELISA titer for each mouse was defined as the highest dilution of serum that resulted in an OD value at least two standard deviations above the average obtained from all negative mouse serum control wells.

### Statistical Analysis

All data were entered into GraphPad Prism 8 for statistical analysis and generation of graphs. All survival curves were compared using a log rank (Mantel-Cox) test. The mixed-effects model with the Geisser-Greenhouse correction and Bonferroni’s multiple comparisons test were used for weight loss comparisons between sexes and over time for each mouse strain. The mixed-effects model with the Geisser-Greenhouse was also run to compare weight data between mouse strains over time. Viral RNA qRT-PCR data were analyzed in Excel. Comparison of viral RNA loads within each tissue for each strain across doses, with the exception of NZO/HILtJ mice, was performed using a one-way ANOVA. For NZO/HILtJ mice, an unpaired *t*-test was used because there were only two challenge doses. Two-way ANOVA, and an overall alpha of 0.05, was used to compare viral RNA loads within each tissue across strains and sex. Viral RNA load in testes was compared between strains using a one-way ANOVA. A *p* ≤ 0.05 was considered statistically significant.

## Results

### Survival and Clinical Observations

**Figure 1:**
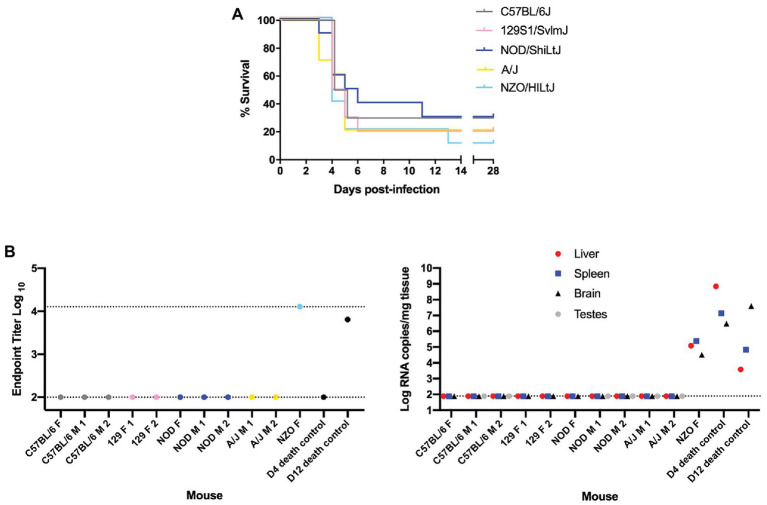
Nearly all “survivor” mice in the 0.2 TCID_50_ challenge group were not infected with Rift Valley fever virus (RVFV). **(A)** Survival curve of five inbred mouse strains infected *via* footpad injection with wild-type RVFV at a dose of 0.2 TCID_50_ shows “survivor” mice across all five strains. Each line represents the percent survival after infection of five female and five male mice. **(B)** Enzyme-linked immunosorbent assay (ELISA) and qRT-PCR analysis of “survivor” mice as well as early and late-death 0.2 TCID_50_-challenged mice as controls. Endpoint RVFV-specific ELISA titer in the serum of mice that survived to day 28 post-infection. Viral load per milligram of tissue in the liver, spleen, brain, and testes of mice that survived to day 28 post-infection was measured by qRT-PCR. The limits of detection for these assays are indicated by dashed lines (ELISA limits of detection due to limited dilutions: lower limit of 100 and upper limit of 12,800; qRT-PCR limit of detection: 77.5 RNA copies/mg).

**Figure 2:**
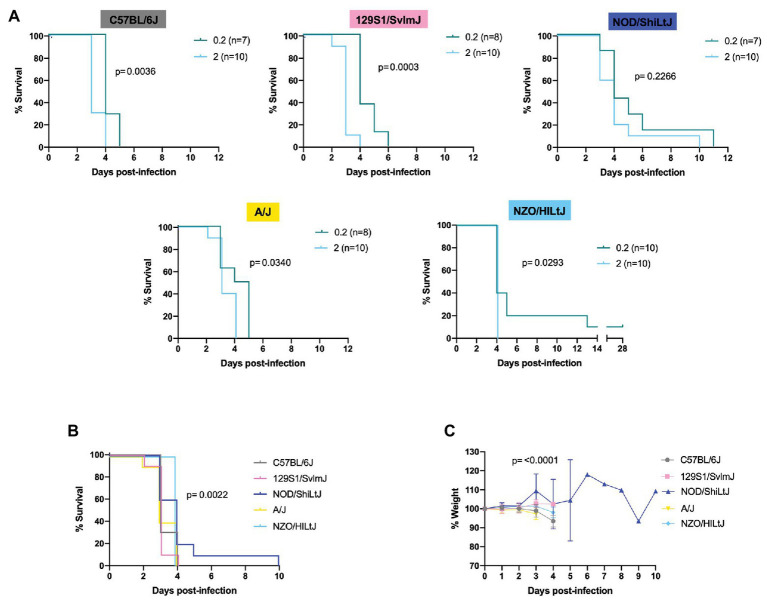
Lethality in five inbred mouse strains following RVFV infection. **(A)** Survival curves of five inbred mouse strains infected *via* footpad injection with wild-type RVFV at doses of 0.2 or 2 TCID_50_ show dose-dependent differences in time to death but not survival. Each line represents the percent survival after infection of female and male mice at a given challenge dose. Confirmed uninfected mice from the 0.2 TCID_50_ dose are excluded from the graphs. **(B)** Percent survival of all five inbred mouse strains when infected with 2 TCID_50_ RVFV. Each line represents the percent survival after infection of five female and five male mice for a given strain. **(C)** Percent change in mouse daily weight from baseline in all five inbred strains after infection with 2 TCID_50_ RVFV. Weight loss curves represent five female and five male mice for a given strain. Survival statistics were calculated using a log rank (Mantel-Cox) test and *p* are marked on all graphs. The mixed-effects model with the Geisser-Greenhouse correction were used for weight loss comparisons between mouse strains over time.

Female and male mice of five genetically inbred mouse strains (C57BL/6J, 129S1/SvlmJ, NOD/ShiLtJ, A/J, and NZO/HILtJ) were infected by footpad injection with doses ranging from 0.2 to 2,000 TCID_50_ of the wild-type ZH501 strain of RVFV. Weight was recorded daily over the course of 28 days and mice were euthanized upon meeting predefined euthanasia criteria, as outlined in Supplementary Table S1, or at the end of the 28 days. Results from initial survival studies showed that RVFV was highly lethal in the evaluated inbred mouse strains, even down to an infection dose of 2 TCID_50_ (Supplementary Figure S1). Therefore, challenge doses were limited to 0.2 or 2 TCID_50_ for subsequent experiments comparing all strains. At the 0.2 TCID_50_ challenge dose, there were mice that survived to day 28 across all five strains (Figure 1A). As would be expected given the probability of delivering a live virus particle to each mouse when administering such a small dose, most of these “survivor” mice at this 0.2 TCID_50_ challenge dose were confirmed to be uninfected by a negative terminal serum ELISA result and negative qRT-PCR of liver, spleen, brain, and testes tissues (Figure 1B). These mice were therefore excluded from subsequent data analysis. The only mouse that lived to the end of the study and was deemed a true survivor was one NZO/HILtJ female mouse. This mouse showed high RVFV-specific terminal serum ELISA titers and detectable tissue viral RNA levels and thus was determined to have been successfully infected at the 0.2 TCID_50_ dose (Figure 1B). This mouse was therefore not excluded from subsequent datasets. Apart from this one survivor, all other mice succumbed to RVFV infection from 2,000 TCID_50_ down to the challenge dose of 0.2 TCID_50_ across all five inbred mouse strains (Figure 2A and Supplementary Figure S1). These results demonstrate the lethality of RVFV down to a single virion in all five of the tested inbred mouse strains. With an LD_50_ impossible to calculate, to the best we can estimate, the LD_100_ of RVFV for all five of the tested inbred mouse strains is 1 infectious virus particle.

Log-rank (Mantel-Cox) tests revealed significant differences in the survival curves between challenge dose groups for all strains except for NOD/ShiLtJ mice (Figure 2A and Supplementary Figure S1). All other strains succumbed to RVFV infection in a statistically significant dose-dependent manner with the 2 TCID_50_ challenge group dying earlier than the 0.2 TCID_50_ challenge group (Figure 2A; Supplementary Figure S1). Across all mouse strains, the median time to death was increased at lower challenge doses. Dose 2 TCID_50_ was the challenge dose that infected 100% of mice while also extending the median time to death by 1 day as compared to higher challenge doses (for example, C57BL/6J mice had a median time to death of 3 days at dose 2 TCID_50_ instead of 2 days at doses 20, 200, and 2,000 TCID_50_). Log-rank (Mantel-Cox) tests resulted in significance between strain survival curves (*p* = 0.0022) at dose 2 TCID_50_ (Figure 2B). However, the difference in time to death between strains is minimal as all inbred mouse strains succumb within 5 days of infection (apart from the one NOD/ShiLtJ female who died at day 10; Figure 2B). Minimal but statistically significant differences in weight loss were observed between strains (Figure 2C). The greatest difference in weight trajectories was the NOD/ShiLt mouse that died of late-onset encephalitis. This mouse lost weight consistently for 4 days, and then exhibited an abrupt increase in weight on the day leading up to its death (Figure 2C).

RVFV-induced death across all five inbred mouse strains tested at a dose of 2 TCID_50_ was consistent with severe hepatic disease. This was evidenced by gross liver pathology including hepatic enlargement and pale foci of necrosis as well as virologic data indicating the liver as the key target of RVFV at the time of death. The only exception to this in the 2 TCID_50_ challenge dose was one late-onset NOD/ShiLtJ death. This mouse cleared most virus from its liver and spleen but died at day 10 of late-onset encephalitis as evidenced by clinical symptoms (tremor) and virologic data. Late-onset encephalitis was also seen in the 0.2 and 20 TCID_50_ dose groups and was the reason for euthanasia for all deaths after day 6. Not all mice showed symptoms of disease before death due to the extremely fast progression of disease. Symptoms, when present before death, were similar among all five inbred mouse strains. Observed symptoms include hunched posture, ruffled fur, piloerection, huddling behavior, eye squinting, and poor response to stimuli. Mice that succumbed or were euthanized past day 6 post-infection displayed symptoms of encephalitis including hind limb paralysis and tremor.

### Sex Differences

**Figure 3:**
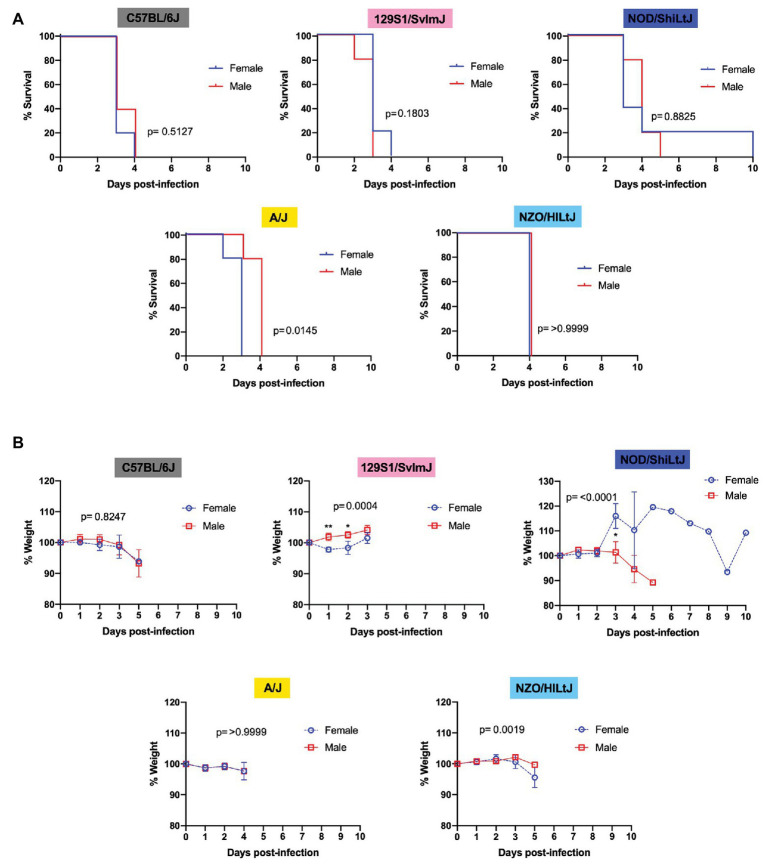
Sex does not impact survival and has only a modest effect on time to death and weight loss in five inbred mouse strains following RVFV infection. **(A)** Survival curves of five inbred mouse strains infected *via* footpad injection with 2 TCID_50_ RVFV show no difference between female and male mice. Each line represents the percent survival after infection of five mice of each sex. **(B)** Percent change in mouse daily weight from baseline after infection with 2 TCID_50_ RVFV. Each weight loss line represents five mice. Survival statistics were calculated using a log rank (Mantel-Cox) test and *p* are marked on all graphs. The mixed-effects model with the Geisser-Greenhouse correction and Bonferroni’s multiple comparisons test were used for weight loss comparisons between sexes and over time for each mouse strain. An asterisk at a particular time point indicates significance (*p* ≤ 0.05) in *post hoc* analysis.

Males and females of all five strains of mice succumbed equally to RVFV infection with 100% lethality at the dose 2 TCID_50_, regardless of sex. Upon comparing female and male mice within each inbred strain using Log-rank (Mantel-Cox) tests, a significant difference in survival curves was only found for A/J mice, with males dying approximately 1 day later than females (Figure 3A). For all other strains (C57BL/6J, 129S1/SvlmJ, NOD/ShiLtJ, and NZO/HILtJ) no significant sex-dependent difference was observed between survival curves (Figure 3A). Interestingly, only 129S1/SvlmJ and NOD/ShiLtJ mice displayed even a slight sex-specific significant difference in weight loss while showing no sex-specific difference in time to death (Figure 3B). Paradoxically, 129S1/SvlmJ mice and female NOD/ShiLtJ mice actually gained weight leading up to death (Figure 3B). All NOD/ShiLtJ female mice gained weight before death; however, the one mouse that succumbed to late-onset encephalitis in this group gained weight suddenly at day 3 post-infection, and then slowly dropped weight until finally gaining weight on the day leading up to euthanasia at day 10.

### Viral Titers

**Figure 4:**
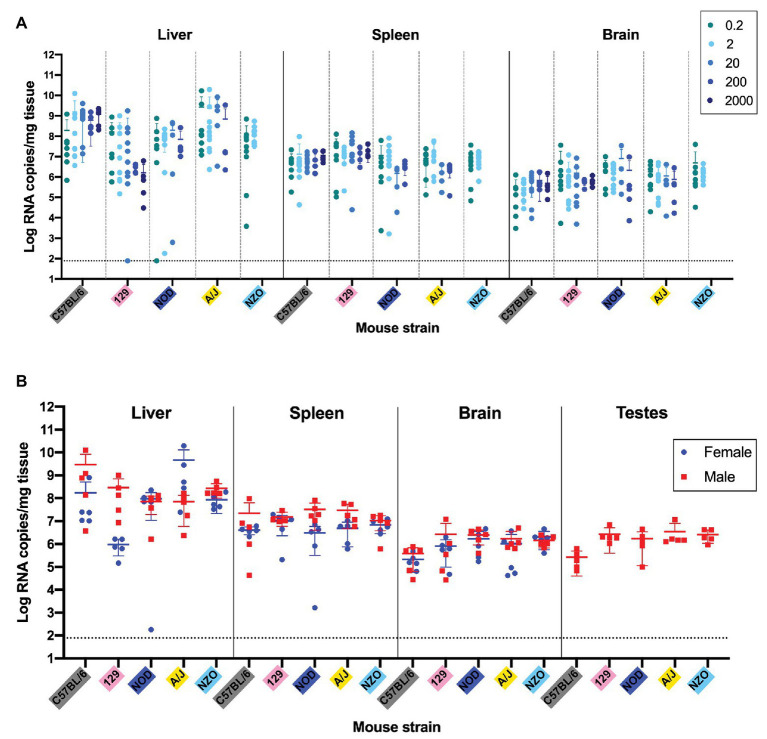
Quantification of viral RNA levels in key tissues of five inbred mouse strains shows no statistically significant dose, sex, or strain differences. **(A)** Viral load per milligram of tissue in the liver, spleen, and brain of mice at the point of death or euthanasia after RVFV infection at doses of 0.2, 2, 20, 200, and 2,000 TCID_50_. Confirmed uninfected mice from the 0.2 TCID_50_ dose are excluded from the graph. **(B)** Viral load per milligram of tissue in the liver, spleen, brain, and testes of mice that succumbed to infection with 2 TCID_50_ RVFV was measured by qRT-PCR. A one-way ANOVA was used to compare viral RNA loads within each tissue for each strain across doses. For NZO/HILtJ mice an unpaired *t*-test was used since there were only two challenge doses. A two-way ANOVA was used to compare viral RNA loads within each tissue across strains and sex. A one-way ANOVA was used to compare viral RNA loads within the testes across strains. The limit of detection of these assays (77.5 RNA copies/mg) is indicated by a dashed line.

Liver, spleen, and brain tissue samples were harvested from all mice for viral RNA level analysis by qRT-PCR. RVFV challenge dose did not significantly affect the viral RNA levels in the liver, spleen, or brain of mice at the time of death/euthanasia (Figure 4A). An assessment of viral load in different tissues as a function of sex or strain was performed using the 2 TCID_50_ dose. This dose was chosen since it uniformly resulted in 100% of the mice being infected. qRT-PCR data are presented as the viral RNA levels in tissues at the time of death for both female and male mice of all five inbred strains (Figure 4B). For male mice, testes were collected to investigate any differences in viral RNA load between strains. There were no significant differences in RNA levels in the liver, spleen, or brain when comparing between the five inbred mouse strains either with sexes combined or separate (Figure 4B). Although there appeared to be sex-specific trends in liver viral load for C57BL/6J, 129S1/SvlmJ, and A/J mouse strains, none are statistically significant by two-way ANOVA (Figure 4B). No significant difference in viral RNA in testes was found between mouse strains when compared using one-way ANOVA. The two low data points that appear on the graph represent the liver and spleen RNA levels from the late-onset NOD/ShiLtJ encephalitis death. The consistency of these RNA load data across both strain and sex are remarkable but logical due to the similarity of disease manifestations and survival timeframes. All strains consistently carry the highest viral load in the liver and the lowest viral load in the brain with the sole exception being the NOD/ShiLtJ mouse that died of encephalitis.

## Discussion

This study evaluated the consequences of RVFV infection in five common inbred laboratory mouse strains: C57BL/6J, 129S1/SvlmJ, NOD/ShiLtJ, A/J, and NZO/HILtJ. RVFV pathogenicity was studied in these strains with challenge doses ranging from 0.2 to 2,000 TCID_50_. Complete lethality was observed in all five inbred mouse strains down to a dose of 2 TCID_50_. This is consistent with other published studies using different strains of inbred mice (do Valle et al., 2010; Smith et al., 2010; Gray et al., 2012). It is not possible to infect a mouse with a portion of a virus particle, therefore, mice in the 0.2 TCID_50_ dose group were either infected with 0, 1, or more virus particles depending on the distribution of virions in the 10 TCID_50_/ml dilution stock. Mice in this dose group that “survived” until day 28 and had negative terminal serum ELISA and tissue qRT-PCR were determined to have not been infected. It is interesting to note that these mice were co-housed with sick and dying animals from the same 0.2 TCID_50_ infection group, revealing a lack of RVFV transmission between animals. This is consistent with the reported absence of person to person transmission (Al-Hamdan et al., 2015). All mice that were successfully infected in the 0.2 TCID_50_ dose group succumbed to disease with the exception of one NZO/HILtJ female mouse that was determined to be a true survivor. From these results, we conclude that one infectious virion is sufficient to cause lethal RVFV disease in C57BL/6J, 129S1/SvlmJ, NOD/ShiLtJ, A/J, and NZO/HILtJ mice.

Throughout the execution of this work consistency in lethality and time to death using a recombinant reverse-genetics generated strain of RVFV was noted. Fully sequence confirmed, early passage recombinant RVFV (rRVFV) strains made through reverse genetics offer far greater reproducibility. This is especially important when evaluating novel mouse strains for their susceptibility to RVFV. Even at a dose of 2 TCID_50_, all mice were successfully infected and time to death was remarkably consistent within each strain. This is likely related to the lack of significant numbers of defective-interfering (DI) particles or viral subpopulations that would accumulate over repeated passaging. In fact, the importance of using recombinant RVFV strains was noted in a recent publication (Ikegami et al., 2017). Ikegami et al. (2017) found that several genetic lineages of RVFV exhibited differences in virulence in outbred CD1 mice. Specific to the ZH501 strain of RVFV, it was found that the ZH501 isolate might contain two distinct viral subpopulations, M847-G and M847-A (Morrill et al., 2010). The two subpopulations were found in a stock of wild-type ZH501 acquired from the CDC, although the authors note that there is no way to determine when the subpopulations arose or if they were present in the infected patient from which the isolate came (Morrill et al., 2010). Infection of mice with the mixture of viral populations was found to be less virulent than the reverse-genetics derived rRVFV M847-A viral variant (Morrill et al., 2010). The increased lethality and reliability obtained by using rRVFV was reflected in our mouse challenge studies.

The inbred mouse strains investigated in this paper were extremely susceptible to RVFV but revealed a significant difference in time to death corresponding to viral dose, with mice challenged at lower doses surviving longer. In this study, the dose of 2 TCID_50_ RVFV was found to be the lowest dose that reliably infects 100% of the tested inbred mice. The 2 TCID_50_ dose was also found to result in the longest median time to death, for C57BL/6J, 129S1/SvlmJ, NOD/ShiLtJ, and A/J mouse strains, as compared to all higher challenge doses. Therefore, this dose allows for the possibility of dilution errors while still challenging mice with at least one virion of lethal RVFV and extends the survival time by 1 day. Doses higher than 2 TCID_50_ were not used to challenge NZO/HILtJ mice; however, it can be hypothesized that this strain would follow the same trend. The acute hepatic inbred mouse challenge models described in this paper, therefore, permit the reliable administration of a low dose of virus, while also consistently extending the intervention window for post-exposure studies or serial euthanasia. These models could be used alongside the highly established BALB/c mouse model, which is undoubtably a useful model for post-exposure studies due to its longer time to death and greater resiliency to RVFV infection. However, time to death in BALB/c mice is varied as well as their course of disease. Therefore, for the study of RVFV hepatic disease, the presented five inbred mouse models represent a consistent model for pathogenesis and intervention studies.

Interestingly, RVFV challenge dose did not affect viral RNA load in mouse tissues at the time of death or euthanasia. This result is comparable to work performed in rats showing no correlation between the dose of administered virus and the amount of infectious virus (PFU) at the time of death (Bales et al., 2012). A lower challenge dose of virus could be slower at establishing infection; however, a maximal viral set point is reached by the time of death/euthanasia regardless of challenge dose. Serial sacrifice studies at early time points post-infection would likely reveal initial differences in viral establishment between infection doses.

Mice from all five strains died of disease consistent with severe acute hepatitis, typically within 5 days of infection at a dose of 2 TCID_50_. Clinical symptoms, gross liver pathology, virologic level trends in key tissues, and time to death were all consistent with the acute RVFV hepatic disease phenotype previously described in BALB/c, C57BL/6J, and MBT mouse models (do Valle et al., 2010; Smith et al., 2010; Gray et al., 2012; Lathan et al., 2017). The only other disease manifestations observed in the infected mice were hind limb paralysis and tremor in the three mice that died after day 6 in the 0.2 and 2 TCID_50_ dose groups. These mice displayed clear encephalitic disease with viral titers appearing highest in the brain while viral RNA was nearly cleared from all other organs, mirroring RVFV encephalitic presentation in other mouse models (Smith et al., 2010; Reed et al., 2013).

It is known that genetic variability exists between commonly used laboratory inbred mouse strains and that these genetic differences have substantial influence on mouse phenotypes. These genetic differences are also present between the strains used in this study and influence such things as the skewing of C57BL/6J mice toward a Th1 response, the development of polygenic obesity in NZO/HILtJ mice or the predisposition of NOD/ShiLtJ mice to develop type 1 diabetes. However, it is clear that the five inbred mouse strains used in this study do not contain adequate genetic diversity to elicit increased resistance to RVFV infection or a skewing toward different manifestations of RVF disease. The one exception to the universal death caused by RVFV in this study was a female NZO/HILtJ mouse that survived to the end of the study and was determined to have been successfully infected from a dose of 0.2 TCID_50_. Although this mouse did not show symptoms or die by day 28, it is possible that it would have died of late-onset encephalitis if the study had been prolonged. Although not a direct comparison, encephalitis deaths in CD4-depleted inbred mice have been seen as late as 36 days post-infection with ∆NSs RVFV (Dodd et al., 2013). However, it is also distinctly possible that this mouse did survive productive wild-type RVFV infection. It is interesting to note that NZO/HILtJ mice are the only strain in this study with a fully functional Mx1 locus (human homologue MxA), which has been shown to have antiviral activity against multiple viruses (Staeheli et al., 1988; Frese et al., 1996; Ferris et al., 2013; Verhelst et al., 2013; Leist et al., 2016). However, as only one out of all challenged NZO/HILtJ mice survived, it is clear that Mx1 is not the main host genetic factor responsible for mouse susceptibility to RVFV.

It is widely accepted that sex significantly influences infectious disease pathogenesis and severity across multiple species (vom Steeg and Klein, 2016, 2017). Males and females of numerous species differ in disease severity to various viral pathogens due to differences in their hormone environments and immune response potentials (Klein and Roberts, 2010, 2015). These sex influences on viral disease severity are complex and thus must be investigated for each individual pathogen. Although in general males are more susceptible to infection by viral pathogens, there are viruses, such as human Papillomavirus and Influenza A virus, that do not follow this rule and are instead higher risk infections in females (Chan et al., 1989; Lorenzo et al., 2011; Hoffmann et al., 2015; vom Steeg and Klein, 2016). However, little data exist on the importance of sex in the context of RVFV infection in mice. Historic RVFV studies have used both sexes of mice, but few have directly compared females and males in the same study.

In this study, both females and males of the five inbred mouse strains were challenged with the same doses of RVFV. There was no significant difference in survival curves or time to death between female and male mice except for a 1-day difference in time to death for A/J mice. Although weight loss was found to be statistically different between sexes for 129S1/SvlmJ, A/J, and NZO/HILtJ mice, this is not felt to be of clinical importance given the overall lethality. Additionally, viral RNA load in the liver, spleen, and brain was not found to be significantly different when comparing females and males of any of the five inbred mouse strains. This failure to identify any significant sex difference in percent survival or viral burden and the extremely modest influence of sex on time to death and weight loss is qualified by the relatively low number of animals challenged in these studies. Five mice per group are not of sufficient power to conclude a total absence of sex differences in RVFV infection. However, even with small sample sizes, the almost uniform lethality seen in both sexes suggests that it would be a reasonable approach to use only one sex for initial survival studies in future experiments, saving both time and resources. In the context of vaccine and therapeutic studies, however, it would still be advisable to evaluate both sexes for potential subtle variation in responses that could be revealed with a more highly powered study.

These data differ from that presented by Tokuda et al. (2015) who found that sex influenced susceptibility to RVFV in MBT (*n* = 90) and BALB/c (*n* = 96) mice as determined by log-rank tests of survival curves. The authors found that males were more susceptible to infection than females, concluding that MBT susceptibility is controlled by multiple genes, of which sex is a contributing factor (Tokuda et al., 2015). Generally, differences in the amount of influence attributable to sex between ours and other’s research could be caused by a variety of factors. These include the mouse strains used, the age of the mice, the RVFV challenge strain, the power of the study, and whether reverse-genetics derived virus or passaged isolate is used for challenge. Additionally, environmental factors between research facilities could affect such things as the mouse microbiome, which has been shown to influence severity of various infectious diseases (Villarino et al., 2016; Li et al., 2019; Robinson et al., 2019).

In summary, C57BL/6J, 129S1/SvlmJ, NOD/ShiLtJ, A/J, and NZO/HILtJ mice are highly susceptible to lethal RVFV infection. In this study, the dose of 2 TCID_50_ wild-type RVFV was found to be the lowest dose that reliably infected 100% of inbred mice tested. All inbred mice that were tested uniformly died of 2 TCID_50_ RVFV challenge, regardless of sex or inbred strain. No substantial differences in disease phenotype or susceptibility were found between the five chosen inbred mouse strains as most died of acute hepatic disease. Therefore, all of these models could be useful for the study of acute lethal hepatitis caused by RVFV. From these studies, it is clear that the five chosen mouse strains do not contain adequate genetic diversity to identify increased resistance to RVFV in mice. Additional studies aimed at assessing mice for divergent RVFV phenotypes that better recapitulate human disease could focus on mice with additional genetic diversity.

## Data Availability Statement

All datasets presented in this study are included in the article/Supplementary Material.

## Ethics Statement

The animal study was reviewed and approved by the University of Pittsburgh IACUC (protocol number 19044158).

## Author Contributions

AM: conceptualization, methodology, investigation, writing and review, and funding acquisition. HC and DB: investigation and manuscript writing and review. All authors contributed to the article and approved the submitted version.

### Conflict of Interest

The authors declare that the research was conducted in the absence of any commercial or financial relationships that could be construed as a potential conflict of interest.
