# Supplementary material for: Rift Valley Fever Virus Is Lethal in Different Inbred Mouse Strains Independent of Sex
Source: Front Microbiol. 2020 Aug 21;11:1962. doi: 10.3389/fmicb.2020.01962 (PMC7472459; doi:10.3389/fmicb.2020.01962)
Supplement: Supplementary file 1 [file Data_Sheet_1.PDF]

Table S1: RVFV-specific mouse clinical scoring system

Clinical Symptom	Score<br>*Scores ≥ 10 will result in immediate euthanasia
• Hunched back<br>• Ruffled coat/piloerection<br>• Huddling	2 points each
• Ataxia<br>• Circling<br>• Weakness<br>• Shaking<br>• Tremors<br>• Dehydration (eye recession)	3 points each
• Anemia (pale mucous membranes, pale footpads)<br>• Abnormal breathing (dyspnea, tachypnea, rales/audible breaths)	5 points each
• Hemorrhage/bleeding<br>• Paralysis<br>• Moribund<br>• Unresponsive	10 points each
• >20% weight loss	Immediate euthanasia
