# Supplementary material for: Rift Valley Fever Virus Is Lethal in Different Inbred Mouse Strains Independent of Sex
Source: Front Microbiol. 2020 Aug 21;11:1962. doi: 10.3389/fmicb.2020.01962 (PMC7472459; doi:10.3389/fmicb.2020.01962)
Supplement: Supplementary file 2 [file Data_Sheet_2.PDF]

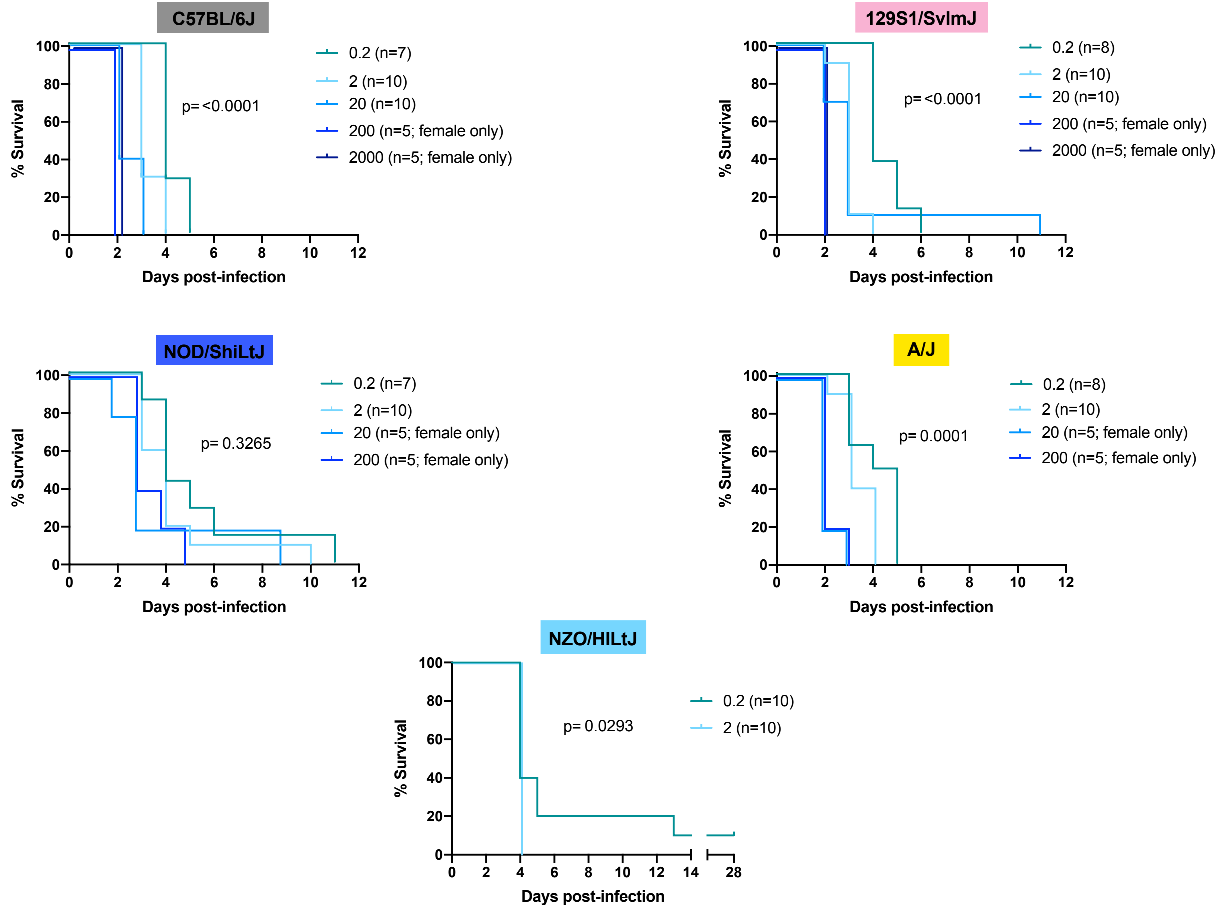

Figure S1. RVFV challenge dose affects time to death in five inbred mouse strains. Survival curves of 5 inbred mouse strains infected via footpad injection with wild-type RVFV at doses of 0.2, 2, 20, 200, or 2000 $TCID_{50}$ show dose-dependent differences in time to death but not survival. Each line represents the percent survival after infection of mice at a given challenge dose. Confirmed uninfected mice from the 0.2 $TCID_{50}$ dose are excluded from the graphs. Survival statistics were calculated using a log rank (Mantel-Cox) test and P values are marked on all graphs.
